# Polymorphism-specific PCR enhances the diagnostic performance of American tegumentary leishmaniasis and allows the rapid identification of *Leishmania* species from Argentina

**DOI:** 10.1186/1471-2334-12-191

**Published:** 2012-08-15

**Authors:** Jorge D Marco, Paola A Barroso, Tatsuyuki Mimori, Fabricio M Locatelli, Ayako Tomatani, María C Mora, S Pamela Cajal, Julio R Nasser, Luis A Parada, Taketoshi Taniguchi, Masataka Korenaga, Miguel A Basombrío, Yoshihisa Hashiguchi

**Affiliations:** 1 Instituto de Patología Experimental, Facultad de Ciencias de la Salud, Universidad Nacional de Salta / Consejo Nacional de Investigaciones Científicas y Técnicas, Salta, Argentina; 2 Department of Parasitology, Kochi Medical School, Kochi University, Nankoku, Japan; 3 Department of Microbiology, School of Health Sciences, Kumamoto University, Kumamoto, Japan; 4 Division of Molecular Biology, Science Research Center, Kochi University, Nankoku, Japan; 5 Instituto de Investigaciones en Enfermedades Tropicales, Sede Regional Orán, Universidad Nacional de Salta, San Ramón de la Nueva Orán, Salta, Argentina; 6 Prometeo Proyecto, Centro de Biomedicina, Universidad Central del Ecuador, Quito, Ecuador

**Keywords:** Leishmaniasis, Tegumentary leishmaniasis, *Leishmania braziliensis*, *Leishmania guyanensis*, *Leishmania panamensis*, PS-PCR, Diagnosis, Argentina, Species identification, *Leishmania* strains

## Abstract

**Background:**

The diagnosis of the leishmaniases poses enormous challenges in Argentina. The Polymorphism-Specific PCR (PS-PCR) designed and validated in our laboratories has been proven effective for typifying the *Leishmania* genus from cultured material. Here we evaluated the performance of this method in the diagnosis of American tegumentary leishmaniasis (ATL) and the rapid identification of *Leishmania* spp. directly from clinical specimens.

**Methods:**

A total of 63 patients from northwestern Argentina, with cutaneous or mucocutaneous lesions, underwent an ATL diagnosis protocol which included clinical examination, Leishmanin skin test, and microscopic examination of dermal smears. In addition, we performed PS-PCR on DNA directly extracted from the specimens scraped from the lesions.

**Results:**

Out of the 63 patients, 44 were classified as ATL cases and 19 as non-ATL cases. The diagnostic sensitivity of the microscopic analysis of dermal smears and PS-PCR individually were 70.5% and 81%, respectively. When performing both tests in parallel, this parameter increased significantly to 97.6% (p = 0.0018). The specificities, on the other hand, were 100%, 84.2%, and 83.3% for the combination, respectively (p > 0.05). Using the PS-PCR analysis we successfully identified the *Leishmania* spp. in 31 out of the 44 ATL cases. Twenty-eight (90.3%) cases were caused by *L. (V.) braziliensis*, two (6.5%) by *L. (V.) guyanensis*, and one (3.2%) by *L. (V.) panamensis*.

**Conclusions:**

The efficacy of the ATL diagnosis was significantly improved by combining the dermal smear examination with a PS-PCR analysis. Our strategy allowed us to reach the diagnosis of ATL with high accuracy regarding the species of the etiological agent in 70.5% of the cases. Moreover, we diagnosed two cases of the disseminated cutaneous form caused by *L. (V.) braziliensis* and a cutaneous case due to *L. (V.) panamensis* infection, both findings reported for the first time in Argentina.

## Background

The leishmaniases are a group of neglected tropical diseases caused by flagellated parasites of the genus *Leishmania*. According to the World Health Organization (WHO), they remain as category I TDR diseases, signifying their status as emerging and uncontrolled, and highlighting the need for new and better tools for their diagnosis, treatment and prevention [1].

American tegumentary leishmaniasis (ATL) is endemic in Argentina with an estimated incidence of 8.76 cases/year/10^6^ inhabitants, calculated from 1984 to 2005 [2]. However, these data might not be accurate since the laboratory resources for confirming ATL are not always available, and local physicians have to report suspected cases merely based on clinical evidence without any diagnostic test. Furthermore, the visualization of amastigotes in smears prepared with material scraped from lesions, requires highly trained personnel, is time consuming, and often shows low sensitivity [3]. In the north of Salta province, where 53.1% of all cases reported in the Argentina occur, some institutions enforce applying the Montenegro skin test (MST) together with microscopic examination of smears to diagnose ATL. Although MST detects past infections or previous contact with the parasite, it is a useful tool for screening or as a complementary test for the diagnosis of an active disease [4].

*Leishmania* species identification, very important for prescribing the appropriate treatments and evaluating the prognosis, is mainly performed for research purpose, and is rarely applied in clinical practice in Argentina. In a previous work, applying multilocus enzyme electrophoresis (MLEE), we determined that *Leishmania (Viannia) braziliensis* was the prevalent species causing three different clinical forms of ATL: cutaneous (CL), mucocutaneous (MCL), and recurrent cutaneous leishmaniasis in Salta province. *Leishmania (Leishmania) amazonensis* and *L. (V.) guyanensis* were also isolated from patients with CL from this area, however only few isolates were specifically characterized so far [4,5].

Considering the presence of several dermatological diseases with similar clinical manifestations to ATL, the reduced applicability of serological diagnostic methods because of their cross reactivity with American trypanosomiasis [6] , and the presence of at least three *Leishmania* spp. in the area, techniques based on the DNA analysis have been developed as alternative approaches for the diagnosis of ATL and the typing of the *Leishmania* genus [7]. Among them, the polymorphism-specific PCR (PS-PCR) has been established in our laboratories for the identification of the five main species responsible for ATL [8]. Moreover, this procedure was validated against MLEE, the gold standard method, using *Leishmania* isolates from local patients cultured *in vitro*[9].

In the present study we evaluated the performance of a modified PS-PCR approach applied directly on clinical samples. Our results demonstrate that the method readily permits the detection and species identification of parasites causing ATL in Argentina. Furthermore, we believe that using this approach might have an impact on the clinical-epidemiological management of patients with ATL.

## Methods

### Ethics statement

All patients voluntarily requested the tests for the differential diagnosis of their lesions, and consented to participate anonymously in this study. The procedures were approved by the Bioethics Committee of the Health Ministry of Salta, Argentina, and followed the Declaration of Helsinki Principles.

### Patients and diagnosis

Sixty-three patients with cutaneous or mucocutaneous lesions suspected of leishmaniasis were included in this study. Patients were diagnosed in three different institutions; the Research Institute of Tropical Diseases, University of Salta, Oran; the Department of Dermatology, Milagro Hospital; and the Department of Dermatology and Otorhinolaryngology, San Bernardo Hospital. All of them are located in Salta, Argentina.

The diagnosis of ATL was performed using a combination of the three criteria currently applied in this area, and described as follows:

#### Parasitological analysis

Search for *Leishmania* amastigotes was done on smears of dermal scrapings from the border of the lesions, obtained with sterile wooden toothpicks, stained with May-Grunewald Giemsa and examined under a microscope as described previously [10].

#### Montenegro skin test

Leishmanin antigen was prepared from a culture of *L. (V.) braziliensis* strain MHOM/AR/03/OLO1, following previously described protocols [4,5]. Four μg (100 μl) of total promastigotes proteins were injected intradermally on the ventral forearm of the patients. Readings were taken 48 hours after the injections, and indurations measuring ≥ 5 mm in diameter were considered reactive [4].

#### Clinical features

The inclusion criterion was the presence of compatible tegumentary injuries, which included ulcerative, nodulous, papulous cutaneous or mucocutaneous lesions of two or more weeks of evolution, and a congruent epidemiological history. Previous diagnoses of CL were taken into account for the diagnosis of secondary MCL cases.

All patients diagnosed in the present work as ATL cases were systemically treated with 10–20 mg d^−1^ kg body wt^−1^ of pentavalent antimony over 25–30 days. In the cases of incomplete clinical cure, another treatment-cycle of the same extension with antimony or amphotericin B was given. The treatments and clinical follow-up were conducted by local physicians.

### DNA samples for PCR assays

Specimens were obtained by scrapping the edge of the lesions with sterile wooden toothpicks and placed in a tube containing 200 μl of TE buffer. The material was heated in a waterbath at boiling temperature for 10 min and then stored at −20°C [11]. DNA was extracted with a phenol-chloroform mixture, precipitated with ethanol and dissolved in 20 μl of TE buffer. A titration assay showed that a 1/20 DNA dilution was optimal for the PS-PCR analyses. Furthermore, the detection limit of the reaction was estimated in 0.3 ng/μl of total *Leishmania* DNA.

Four *Leishmania* strains, MHOM/AR/03/OLO1 of *L. (V.) braziliensis*, MHOM/AR/99/JDM1 of *L. (V.) guyanensis,* MHOM/PA/71/LS94 of *L. (V.) panamensis,* and MHOM/BR/73/M2269 of *L*. *(L.) amazonensis*, were used as controls. Their DNA was extracted from promastigotes cultures according to described protocols [5,12], and purified using the GenomicPrep Cell and Tissue DNA Isolation Kit (Amersham Biosciences, NJ) following the manufacturer’s instructions. The DNA concentrations were determined in a NanoDrop ND-1000 spectrophotometer (NanoDrop Technologies, Wilmington, DE), and adjusted to 10 ng/μl with Milli Q water.

### Polymorphism-specific PCR

This analysis took into account of the genomic differences among species of the *Leishmania* genus. We first performed Arbitrarily-Primed-PCR on total DNA from the five most prevalent species associated with ATL and sequenced those DNA fragments present in single species to design the primers addressing these polymorphisms. In addition, the PCR conditions were adjusted increasing the stringency in order to have the necessary specificity for each reaction [8]. The reactions were performed in duplicate in a GeneAmp PCR System 2400 (Perkin Elmer, Wellesley, MA) using the Roche GeneAmp XL PCR Kit (Applied Biosystems, Foster City, CA) in 15 μl final volume.

The PS-PCR procedure involved two steps; the first was aimed at the ATL diagnosis and also the identification of *Leishmania* subgenus or *Leishmania* complexes. It consists of two reactions, A and B, performed simultaneously in two separate tubes. Reaction A was done with the primers V1-V2, specific for the *Viannia* subgenus (Table 1), under the conditions previously reported [9]. Reaction B was performed with the primers M1-M2 for the detection of *L. mexicana* and *L. donovani* complexes belonging to the *Leishmania* subgenus [13], under the following conditions: initial denaturation at 94°C for five minutes, 35 cycles (30 sec at 94°C, one minute at 60°C, one minute at 72°C), and a final extension step at 72°C for seven minutes) [14]. 

**Table 1:** Polymorphism-Specific PCR. Primer sequences and expected size of amplicons

	**Sequence**		**Amplicon size**
**Primers**	**Forward**	**Reverse**	**(bp)**
V1– V2	5’GCTTCTCGTTTCGCTTTGAAC3′	5′-CAAGACAAGAAAAAAGGCGGC-3′	168
M1– M2	5′CCAGTTTCGAGCCCCGGAG-3′	5′-GGTGTAAAATAGGGGCGGATGCTCTG-3′	700
b1-b2	5′-GTGGGCGTATCTGCTGATGAC-3′	5′-CAAAAAGCGAGGGACTGCGGA-3′	103
p1-p2	5′-GGTCGGATCTGCATGCATCAC-3′	5′-CAAAAAGCGAGGGACTGCGGG-3′	79
g1-g2	5′-GGTCGGATCTGCATGCATCAT3′	5′-CAAAAAGCGAGGGACTGCGGG-3′	79
GAPDH	5′-CGGGAAGCTTGTGATCAATGG-3′	5′-GGCAGTGATGGCATGGACTG-3′	862

The second step addressed the identification of *Leishmania* species. The reactions were performed with three different sets of primers, b1-b2 for *L. (V.) braziliensis*, p1-p2 for *L. (V.) panamensis* and g1-g2 for *L. (V.) guyanensis* on DNA which gave the expected products with primers V1-V2, under conditions previously reported [9].

If a sample resulted positive for reaction B, it was assayed again with the same M1-M2 primers, but at 67.5°C or 72°C annealing temperature, which are specific for *L. mexicana* complex and *L. (L.) amazonensis*, respectively [14].

DNA extracted from *Leishmania* promastigotes cultured *in vitro* or from confirmed ATL patients was used as a positive control, whereas DNA of eight healthy individuals from non-endemic area (Japanese subjects) or from lesions of non-ATL patients was used as negative control.

Additional experiments to control the DNA preparation procedure, as well as to detect the presence of PCR inhibitors were performed using the TaKaRa Ex Taq DNA polymerase Hot Start Version (Takara-Bio, Shiga, Japan) and specific primers for the human glyceraldehydes 3-phosphate dehydrogenase (*GAPDH*) gene, following a previously reported protocol [15].

The PCR products were separated in agarose gels containing ethidium bromide, visualized under UV light and the results were recorded with a Kodak EDAS 290 gel documentation system (Kodak, Rochester, NY).

### Statistical analysis

The diagnostic performance of the tests alone or combined was assessed by calculating several statistical indicators, including sensitivity, specificity and predictive values. First the patients were classified into two groups: ATL cases and non-ATL cases based on the presence or absence of the disease. Then the results of the PS-PCR method for each patient were added to the tables and analyzed independently of the ATL group definition. The sensitivity, specificity, predictive values, and the Cohen’s kappa coefficient were estimated applying Epidat 3.1 software (Pan American Health Organization, Spain) [16]. The predictive values for the laboratory were calculated by applying the Theorem of Bayes assuming a prevalence of 54.82% for the year 2005 with the same software. The proportions were compared by Statistic-*z* analysis or the Fisher’s exact test [16]. The positive and negative PCR controls, or results from non-endemic healthy controls were not included in any of these calculations.

## Results

**Figure 1:**
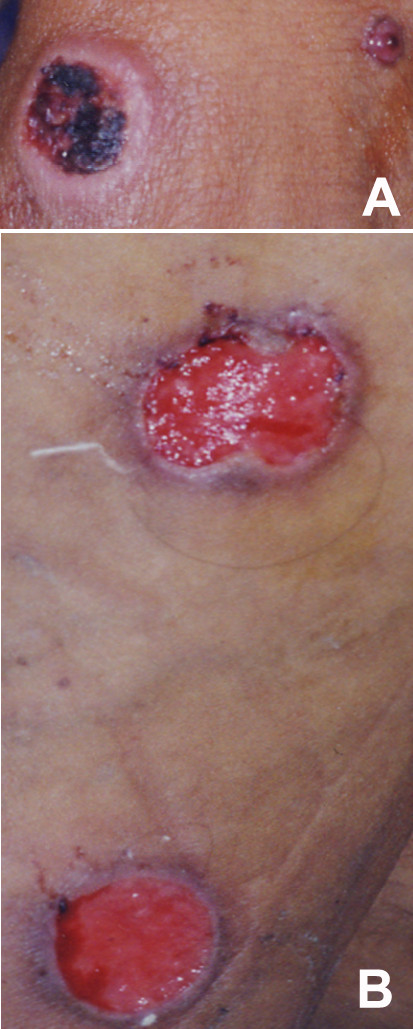
**Disseminated cutaneous*leishmaniasis*.** A patient with multiple ulcerated lesions of different degrees of evolution found on hands (**A**) and legs (**B**). *Leishmania* amastigotes were found in smears from the most recent lesions located on hands, face and the back of the patient. The lesions present in legs were the oldest. The Montenegro skin test was reactive, and *L. (V.) braziliensis* was incriminated as the causative agent by PS-PCR.

The study included 63 patients; eight females (12.7%) and fifty five males (87.3%), with a mean age of 39.6 ± 17.4 years (mean ± standard deviation). High variability in terms of the number, size and evolution-time was observed, skin-lesions measured 2.9 ± 1.7 cm in diameter, and evolved over 2.2 ± 1.8 months, while the progression-time of lesions located on oropharyngeal mucosa was 26.4 ± 22.8 months. Regarding to their distribution, in 30 patients (47.6%) skin lesions were located on the extremities, and in 24 subjects (38.1%) they were located on the head, including 11 (17.5%) patients with mucocutaneous injuries. Two patients (3.2%) had lesions on the trunk. In addition, five patients (7.9%) presented lesions on two different parts of their bodies, and two patients (3.2%) had more than 30 lesions distributed all over their bodies, including the oropharyngeal mucosae (Figure 1). Out of 43 patients who stated their employment situation during the clinical examination, 24 (55.8%) were rural workers, six (13.9%) were unemployed and four (6.3%) were housekeepers.

The diagnosis of ATL was based on the clinical features, the parasitological analysis, and the results of MST, applied simultaneously to each patient. The parasitological method was considered the gold-standard, meaning that the visualization of *Leishmania* amastigotes in the smears indicates presence of the disease. Out of the 63 patients with cutaneous or mucosal lesions, 44 (69.8%) were diagnosed as ATL-cases, whereas the remaining 19 were classified as non ATL-cases, the group representing absence of the disease (Table 2). It is noteworthy that patients with negative parasitological examinations were considered as ATL-cases only if they presented a reactive MST in addition to consistent clinical features and a congruent epidemiological history. Thus, the 19 patients who formed the non-ATL group shared negative results for both parasitological and MST methods.

**Table 2:** Results of smear and PS-PCR individually, or combined in parallel, applied to 63 patients

		**ATL**		
**Assay**	** Result**	**Presence**	**Abscence**^**1**^	**Total**
Smear	Positive	31	0	31
	Negative	13	18	31
	Total	44	18^2^	62
PS-PCR	Positive	34	3	37
	Negative	8	16	24
	Total	42^3^	19	61
Smear + PS-PCR	Positive	41	3	44
	Negative	1	15	16
	Total	42	18	60

ATL = American tegumentary leishmaniasis. ^1^Determined by the criterion mentioned in text. ^2^One out of 19 non-ATL cases was not considered for the analysis because cells were not observed in the smear. ^3^Insuficient DNA was obtained from two samples out of 44 ATL cases.

### Performance of PS-PCR in the diagnosis of American tegumentary leishmaniasis

**Figure 2:**
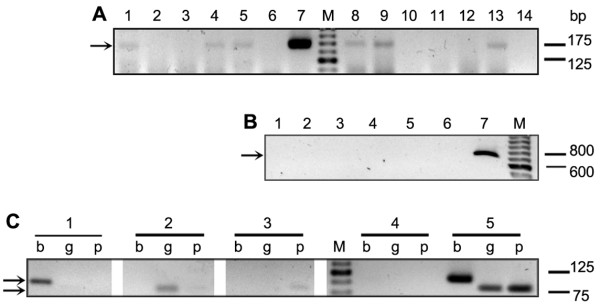
**PCR-based diagnosis and*Leishmania*species assignation.** PCR products from 12 samples from patients using V1-V2 primers for *Viannia* subgenus identification. Lanes 1, 4, 5, 8, 9 and 13 were positives, and lanes 2, 3, 10, 11, 12 and 14 were negatives. Lanes 6 and 7 are the negative and positive control, respectively (**A**). PCR using M1-M2 primers for *L. mexicana* and *L. donovani* complexes applied to five DNA samples. Lanes 1 to 5 were negatives. Lanes 6 and 7 negative and positive control, respectively (**B**). Species identification by PCR analysis with primers b1-b2 (b), g1-g2 (g) and p1-p2 (p) specific for *L. (V.) braziliensis, guyanensis* and *panamensis*, respectively (**C**). Patient 1: Lane b = positive, g and p = negatives. Patient 2: g = positive, b and p = negatives. Patient 3: p = positive, b and g = negatives. Lanes 4 and 5, negative and positive control, respectively. M: marker of molecular weight. Arrows indicate the expected location of the bands.

DNA extracted from the material scrapped from the lesions of the 63 patients was subjected to PS-PCR analysis. Primers V1-V2 were used for the identification of the *Viannia* subgenus and M1-M2 for *L. mexicana* and *L. donovani* complexes. The analysis with primers V1-V2 showed that 34 ATL patients were infected with parasites belonging to the *Viannia* subgenus (Table 2, Figure 2A). Whereas PCRs with primers M1-M2 did not yield any amplification product at 60°C (Figure 2B), an annealing temperature proved effective in detecting all species of the *Leishmania* subgenus responsible for ATL or New World visceral leishmaniasis [14]. 

Amastigotes were observed in 31samples from ATL patients (Table 2), whereas PS-PCR demonstrated the presence of parasites in 34 samples. By combining both methods we were able to accurately diagnose 41 out of the 44 ATL cases. These results were used to calculate indicators of the diagnostic performance shown in Table 3. Microscopic detection of parasites possesses, by definition, the highest diagnostic specificity, whereas the molecular method exhibits more sensitivity than the former. Interestingly, the combination of approaches in parallel increased substantially the diagnostic sensitivity. It is noteworthy that a positive result for the combination implies that either the smear or PS-PCR was positive, whereas a negative result implies negativity with both of them [16]. Statistical analysis also demonstrated that there is a fair agreement between PS-PCR and smear (Cohen’s kappa coefficient = 0.237 ± 0.113). 

**Table 3:** Performance of smears and PS-PCR analyses, and their combination, in the diagnosis of ATL

**Assay**	**Sensitivity (%)**	**Specificity (%)**	**PPV (%)**	**NPV (%)**
Smear	70.5	100	99.9	73.6
PS-PCR	81	84.2	86.2	78.5
Smear + PS-PCR	97.6^1^	83.3	87.9	91

PPV = positive predictive values; NPV = negative predictive values, estimated applying the theorem of Bayes. The positive and negative PCR controls and the results from healthy individuals from non-endemic areas were not included in the calculations.^1^The differences between proportions were statistically significant (*P =* 0.0018) when the Smear + PS-PCR were compared against these tests alone.

The PS-PCR has shown a significant higher sensitivity than smears for the diagnosis of MCL. Among nine patients with this clinical form, eight were found positive by PS-PCR, while only two by smear observation (p *<* 0.05).

Control PCRs with primers for the human *GAPDH* gene demonstrated that the sampling and purification steps of the protocol were effective and also that the samples were devoid of PCR inhibitors. However, weak bands were obtained with two out of the 44 samples, most likely due to low DNA concentration. Since these samples did not give conclusive results with V1-V2 and M1-M2 primers, they have not been considered for calculations of the sensitivity and the positive predictive values (Table 2).

### Identification of *Leishmania* species by PS-PCR

The total of 34 samples in which we detected parasites of the subgenus *Viannia* (see above, Table 2) were subjected to PCR analyses using the primers b1-b2, g1-g2 and p1-p2. The identification of *Leishmania* spp. was achieved in 31 out of these 34 samples, which represents 70.5% of all 44 ATL cases studied (Figure 2C). *Leishmania (V.) braziliensis* was identified as the etiological agent in 28 ATL cases, which included the four clinical forms of the disease: single cutaneous leishmaniasis (SCL), multiple cutaneous leishmaniasis (MultCL), disseminated cutaneous leishmaniasis (DSCL), and MCL, whereas *L. (V.) panamensis* and *L. (V.) guyanensis* were implicated in one and two ATL cases, respectively (Table 4).

**Table 4:** ***Leishmania*species identified by PS-PCR causing ATL in NW Argentina**

***Leishmania*spp.**	**Prevalence**				**Total (%)**
	**SCL**	**MultCL**	**DSCL**	**MCL**	
*L. (V.) braziliensis*	15	4	2	7	28 (90.3)
*L. (V.) guyanensis*	-	2	-	-	2 (6.5)
*L. (V.) panamensis*	1	-	-	-	1 (3.2)
L. mexicana or donovani complex	-	-	-	-	0 (−)

ATL = American tegumentary leishmaniasis; SCL = single cutaneous leishmaniasis; MultCL = multiple cutaneous leishmaniasis; DSCL = disseminated cutaneous leishmaniasis; MCL = mucocutaneous leishmaniasis.

The patients with DSCL, caused by *L. (V.) braziliensis*, had more than 30 lesions each, distributed throughout the body, compromising face, abdomen, extremities, back, and oro-nasal mucosae. The lesions showed different degrees of evolution, from the early papular to the predominant ulcerated form, developing within a two month period following the appearance of the single cutaneous lesion (Figure 1). *Leishmania* amastigotes were found in the smears from several lesions and the MST was reactive in all cases. All these patients responded well to the antimonial treatment.

## Discussion

The development and/or improvement of diagnostic tools remain a priority in the research field of leishmaniasis. In particular, much work has to be done for increasing the sensitivity and specificity of the diagnostic tests for ATL in endemic areas, such as NW Argentina [1]. In this work, we assessed both the impact of adding a PS-PCR analysis to the diagnosis-methods currently used in Argentina, and its usefulness to the identification of the causal-agent of the disease at the species level.

Indeed, by performing PS-PCR directly on clinical samples, we enhanced significantly the efficacy of the ATL diagnosis, especially for MCL. Appling this method to patients with negative results for the microscopic search of parasites in dermal smears, allowed us to increase substantially the diagnostic sensitivity from 70.5% for smears alone to 97.6% for the combination (Table 3). Thus, these results confirm that the PCR analysis is a valuable and applicable strategy for these cases of suspected ATL with negative microscopic examination. The relevance of this strategy becomes apparent when considering the availability of effective treatments or the tendency of the disease to evolve into severe clinical forms if left untreated. These factors indicate that the sensitivity of the diagnostic method should have the priority for maximization, rather than its specificity [17]. Previous PCR analyses for the diagnosis of leishmaniases displayed sensitivities ranging from 51.7% to 100% [18-20]. This wide variability in the sensitivity may very well be due to the methods employed, but also attributable to differences in the sampling techniques, or the clinical and laboratory criteria applied for defining the presence or absence of the disease [7]. The sensitivity of the PS-PCR analysis estimated in this study (81%) is in close agreement with those reported by other investigators when the method was performed on samples from cutaneous lesions of patients with ATL from endemic areas of Ecuador [21,22]. Among the reasons for false negative PS-PCR results, the low parasite burden observed in the borders of the lesions might be of importance. Therefore, improving the sampling method, for example, using a syringe to aspirate the sample or collecting the exudates after scraping the lesions, in addition to efficient DNA extraction, would appear to substantially increase sensitivity and diagnostic efficacy [22].

In the present work, three non-ATL cases were PS-PCR positive, lowering the specificity to 84.2%. Since these patients presented atypical lesions, and were negative for the microscopic parasitological examination and the MST test, we think that these results were due to contamination of the DNA sample.

An identification of *Leishmania* spp. with PS-PCR applied directly on material scrapped from the lesions was achieved in 70.5% of ATL cases*. Leishmania (V.) braziliensis* was the most prevalent species (90.3%) among ATL patients, and this is consistent with previous studies aimed to characterize *Leishmania* stocks isolated in NW Argentina [5,23]. Since we performed the analysis on samples collected directly from the lesions, the indicators of species prevalence reported here might be more accurate than those published before. Moreover, the method has additional advantages, as it gives the *Leishmania spp.* identification without further procedures, such as digestion analysis of the PCR products or gene sequencing, and it is applicable in relatively low-resource laboratories.

*Leishmania (V.) braziliensis* was involved in two cases DSCL reported for the first time in Argentina (Figure 1). Local ATL cases due to *L. (V.) guyanensis* were also found, confirming our previous report on the presence of this species in this area [9]. These findings are of clinical relevance for the patients, because they require a different treatment, such as a protocol with pentamidine rather than the pentavalent antimonials commonly prescribed in this area [19,24]. Interestingly *L. (V.) panamensis* was identified in one sample (Figure 2C). This is the first case reported in Argentina. Although parasites belonging to the *Leishmania mexicana* or *donovani* complexes were found involved in cases of leishmaniasis in this area [4,25], we could not prove their involvement in the present series (Table 4).

## Conclusions

In conclusion, by adding the PS-PCR method to the procedures commonly applied in the country, we improved the diagnostic performance of ATL. The strategy also allowed the identification of *Leishmania* spp. in the vast majority of the cases without isolation of the parasites *in vitro.*

## Competing interests

The authors declare that they have no competing interests.

## Authors’ contributions

JDM, PAB, MK, MAB, and YH conceived and designed the study. JDM, MCM, SPC, and JRN performed the diagnosis and obtained the biological samples. JDM, PAB, TM, FML, AT, and TT carried out the experiments. JDM, LAP, MK, MAB, and YH wrote the manuscript. All authors read and approved the final manuscript.

## Pre-publication history

The pre-publication history for this paper can be accessed here:

http://www.biomedcentral.com/1471-2334/12/191/prepub
